# Intraoperative Assessment of Resection Margin in Oral Cancer: The Potential Role of Spectroscopy

**DOI:** 10.3390/cancers16010121

**Published:** 2023-12-26

**Authors:** Máté Vlocskó, József Piffkó, Ágnes Janovszky

**Affiliations:** Department of Oral and Maxillofacial Surgery, Albert Szent-Györgyi Medical School, University of Szeged, Kálvária 57, H-6725 Szeged, Hungary; vlocsko.mate@med.u-szeged.hu (M.V.); piffko.jozsef@med.u-szeged.hu (J.P.)

**Keywords:** oral cancer, resection margin, intraoperative assessment, spectroscopy

## Abstract

**Simple Summary:**

Several factors may influence the outcome of a patient with cancer, including oral cancer. Modern diagnostic tools have had a great impact on the success of patient care. However, diagnostic methods, even at the moment of surgery, contributing to the development of personalized oncological treatment are indispensable.

**Abstract:**

In parallel with the increasing number of oncological cases, the need for faster and more efficient diagnostic tools has also appeared. Different diagnostic approaches are available, such as radiological imaging or histological staining methods, but these do not provide adequate information regarding the resection margin, intraoperatively, or are time consuming. The purpose of this review is to summarize the current knowledge on spectrometric diagnostic modalities suitable for intraoperative use, with an emphasis on their relevance in the management of oral cancer. The literature agrees on the sensitivity, specificity, and accuracy of spectrometric diagnostic modalities, but further long-term prospective, multicentric clinical studies are needed, which may standardize the intraoperative assessment of the resection margin and the use of real-time spectroscopic approaches.

## 1. Introduction

Every year, approximately 350,000 people worldwide are diagnosed with oropharyngeal cancer [1,2]. Well-known modifiable risk factors, such as smoking and excessive alcohol consumption [3,4,5,6], play an important role, but high-risk human papillomaviruses (HPV) also have a particular impact on the development of oral cancer in younger patients [7,8] (Table 1). Regular screening and knowledge of the risk factors plays an important role in the early diagnosis of oral cancer, while innovation in medicine has contributed to an improvement in the survival rate of patients.

Over the last three decades, significant advancements in diagnostic methods and novelty in complex oncological management, such as targeted therapy and biological therapy, have contributed to the overall 5-year survival rate of patients with oral cancer [9,10,11] (Table 2). Although many factors influence life expectancy (e.g., TNM staging, or comorbidities), the resection margin and depth of invasion are the most important surgical factors that may be compromised to save a specific function or for appearance reasons. The goal of oral cancer surgery is to remove the malignant tissue, while preserving as much healthy tissue and functionality as possible. This requires careful planning and implementation of the surgical procedure, including meticulous identification and removal of the tumor and surrounding margins [12,13].

In general, an adequate resection margin predicts not only the risk of local recurrence, but consequently the survival rate of patients with malignant lesions [14,15,16]. The resection margin is considered negative when it is larger or equal to 5 mm, close to between 1 and 5 mm, or positive when it is less than 1 mm [17,18,19]. Inadequate resection margins lead to higher morbidity and complications, requiring adjuvant treatment, such as radiotherapy, chemoradiation, or re-operation [20].

Several factors can contribute to difficulty in achieving negative resection margins in oral cancer. One of the main problems is the complex and varied anatomy of the oral cavity (e.g., nerves, vessels, or salivary glands), which can influence the extent of the resection, increasing the risk of positive margins. Additionally, inadequate visualization of the tumor or resection margins during surgery can be problematic.

Traditionally, inspection, palpation, and preoperative imaging techniques have been used to determine the resection margin of the tumor, which were supported later by an intraoperative frozen section with the aim of differentiating the tumorous tissue from the healthy tissue. Subsequently, further pathological prognostic factors, such as the “transition zone”, were involved in the comprehensive pathological investigation, where the relationship between the intact and neoplastic cells can be investigated [21,22]. According to “field cancerization theory” within this preneoplastic area, histological and molecular changes may occur, but these changes are not always detectable. Therefore, undetected tumor cells may remain in the operation field [23].

In the present narrative review, the PubMed database was analyzed, including publications published between 1990 and 2023, and the search was conducted between January and October 2023. The terms used in the search included “oral cancer”, “Raman spectroscopy”, “mass spectroscopy”, and “resection margin”. We restricted the search to articles published in English. Because the intraoperative use of different spectroscopic modalities is a relatively new and dynamically developing field in the assessment of the resection margin in oral cancer, we included all types of publications in the search, except case reports. This narrative review presents recent data on the intraoperative assessment of the resection margin in oral cancer and on the potential role of different spectroscopies.

## 2. Contemporary “Imaging Techniques”

Advanced radiological imaging techniques during preoperative planning to achieve a tumor-free tissue resection margin have improved in the last few decades. Standard imaging techniques include magnetic resonance imaging (MRI), computed tomography (CT), panoramic radiography (OPT), positron emission tomography (PET)–CT, and single-photon emission CT (SPECT). These techniques may improve diagnostic or postoperative irradiation accuracy [24,25], but do not provide information about the intraoperative situation.

Different methods (e.g., histological, molecular biological, or spectroscopic modalities) are available to assess the resection margins intraoperatively, but some of them are not possible in real time or applicable in the operating room. Frozen section (FS) diagnosis, as a “gold standard” technique, was used in the early 20th century [26]. Some studies have revealed that FS is unsuitable to determine the resection margin status; as an alternative, a gross examination of the tissue with a 7 mm cut-off should be preferred, which is not a time-consuming or costly method [14,27,28]. Despite the limitations of the FS technique (e.g., suboptimal tissue preparation, cautery artifacts, and/or inadequate sampling, prolonging the operation time) [29], several studies have focused on improving the efficacy of this histological method in the intraoperative assessment of the resection margin (IOARM). In general, specimen-driven (SD) IOARM appeared to be more predictive of the actual margin status than tumor-bed or defect-driven (DD) margins [2,30,31,32,33] and became a standard procedure. Kubik et al. described several reasons (e.g., additional resection at an incorrect location, the incorrect orientation of the additional resection, the incorrect dimensions of the additional resection) for additional resections to be inadequate [34]. In contrast, a study published by Maharaj DD et al. did not reveal a significant difference between SD and DD approaches regarding the sensitivity or specificity of intraoperative FS for resection margin assessment, and similarly for loco-regional recurrence or overall survival [35]. Based on the foregoing, it can be concluded that the sampling protocol, because of cooperation between the surgeon and the pathologist, may lead to significant improvements in the rate of adequate resections, consequently improving the patient outcome and reducing the need for postoperative radiotherapy [33,36,37]. Another recent study focused on deep resection margins and found that there was no significant difference in the recurrence rate between close and clear mucosal margins, while a deep resection margin with residual tumorous tissue was found in 87% of the cases. The most important thing should be to define the optimal depth of the resection margin and to adapt this to the sampling depth of further techniques assessing the resection margin to be developed. Until then, the most prevalent technique remains frozen section analysis [38,39,40]. Although the staged resection technique for skin tumors, Mohs micrographic surgery (MMS), is time consuming and resource intensive for the pathology department, it results in a low recurrence rate in extensive cutaneous oral cancers, but because of the anatomical complexity of this region and the frequent bony involvement, this method cannot be adapted to oro-pharyngeal cancers [41]. Histological assessment informs the surgeon about the margins of the soft tissues, but the intraoperative assessment of bone remains challenging, because it technically cannot be integrated in the limited time frame of the intraoperative margin analysis. Intraoperative cytological assessment of bone resection margins could be a feasible diagnostic tool to verify microscopically the resection margins in bony tissue, showing high sensitivity and specificity in a previous study published by Nieberler et al.; however, the dehydration process can cause altered cell morphologies at the resection margin of the bone [42,43,44]. As published previously, molecular analysis of genetic mutations (e.g., p53) or epigenetic markers (e.g., protein expression or methylation profiles) in surgical margins have a clinical impact, yielding a more sensitive and accurate assessment, and providing insight into their impact on the postoperative prognosis of patients. However, the lack of real-time availability considerably limits their intraoperative use [33,45].

## 3. Spectroscopy for the Intraoperative Assessment of the Resection Margin

Considering the limitations of histological techniques, different forms of IOARM are currently utilized to improve the efficacy of the assessment of tumor resection margins, and to reduce the consecutive operation time related to the examination. Systematic reviews published recently have summarized the opportunities to investigate tumor resection margins [33,40]. Aside from pathological techniques, Kain et al. distinguished two major groups of IOARM: wide- and narrow-field analysis. Wide-field imaging (non-/fluorescent dyes, autofluorescence imaging, and narrow-band imaging) provides real time, intraoperative, visual feedback, but its efficacy may be limited by inflammation and non-malignant processes. Although narrow-field analysis (spectroscopy, optical coherence tomography, confocal microscopy, and high-resolution micro-endoscopy) requires special equipment and training for accurate use, it can provide quantifiable results in the operating room. These techniques are less influenced by inflammatory processes, and some of them can also be used to assess bony structures, adding to their versatility and utility [33]. These controversies have led to the investigation of optical and spectroscopic methods and their utilization as a surgical device for intraoperative use [46,47,48] (Table 3). Spectroscopy, a form of narrow-field analysis, may play a significant role in the intraoperative assessment of resection margins in oral cancer. Unlike histological techniques, spectroscopy offers a non-invasive and real-time approach to evaluating margins during oncological surgery.

Spectroscopy is an analytical technique that has transformed our understanding of the world around us. By measuring the interaction between electromagnetic radiation and matter, spectroscopy provides valuable insights into the chemical composition, molecular structure, and physical properties of materials at the atomic level. This technique has diverse applications across various fields, including materials science, biology, and chemistry. Furthermore, spectroscopy has shown great potential in the medical field, particularly in the detection and characterization of disorders, such as malignant tumors [49,50].

There are several types of spectroscopies, each with its own unique way of probing materials. Infrared spectroscopy, for example, uses infrared radiation to penetrate the surface of a material and explore its molecular vibrations. This technique is commonly used in the analysis of organic compounds and is particularly useful in identifying functional groups in a molecule. On the other hand, Raman spectroscopy utilizes laser light to provide insights into molecular vibrations and crystal structures, making it valuable for the analysis of solids and liquids. The application of different spectroscopies has a wide range of uses, such as in materials science, biology, and chemistry, among others. Medical applications of spectroscopy have also been explored, including in the detection and characterization of disorders, such as malignant tumors or the assessment of tumor resection margins [50,51,52].

In conclusion, spectroscopy holds great potential in the intraoperative assessment of resection margins in oral cancer. By utilizing spectroscopy as part of the IOARM, surgeons have more accurate and efficient means to achieve successful surgical outcomes. While current methods for margin assessment can be laborious, subjective, and logistically demanding, spectroscopy offers a potential solution to overcome these limitations [53]. With further research and development, this technique can revolutionize intraoperative evaluations, providing surgeons with more accurate and efficient means to improve margin assessment and to achieve successful surgical outcomes. Advancements in technology can lead to the development of more portable and user-friendly spectroscopic devices, making them more accessible in the operating room. Additionally, the integration of artificial intelligence and deep learning algorithms can improve the accuracy and efficiency of data analysis, allowing for real-time and automated interpretation of spectroscopic results. These developments can further streamline the intraoperative margin assessment process, reducing the reliance on subjective evaluations and increasing the reliability of the results. With ongoing research and innovation, spectroscopy has the potential to become a standard tool in oncological surgery, improving surgical outcomes and patient care.

### 3.1. Raman Spectroscopy

The first mention of the inelastic scattering of light can be linked to Adolf Smekal in 1923, while it was first observed by Raman in organic liquids and, independently, by Landsberg and Mandelstam in inorganic crystals in 1928. This optical technique provides detailed information about the molecular compounds in the investigated tissue [54,65,66]. Because of the weakness in spontaneous Raman scattering and despite the endeavor to reduce the signal-to-noise ratio, the use of this modality as a diagnostic or intraoperative surgical tool presents some challenges. More than 25 types of Raman spectroscopy (RS) techniques are known, such as spontaneous Raman, hyper-Raman scattering, Fourier transform Raman scattering, Raman-induced Kerr effect spectroscopy, stimulated/coherent Raman scattering, coherent anti-Stokes Raman scattering (CARS), surface-enhanced Raman scattering (SERS), or tip-enhanced Raman scattering (TERS) [67,68].

RS is a promising diagnostic device that can analyze disorders at the molecular level, providing objective, quantifiable information for diagnosis and treatment evaluation in a non-destructive manner. This method is appropriate for tissue and cancer characterization in different regions, such as the central nervous system, the urogenital, or the gastrointestinal tract; however, it is essential that more comprehensive Raman spectral databases and tissue classification methodologies are developed to ameliorate its clinical applicability [69,70,71,72]. In the head and neck region, RS can also be used to differentiate oral squamous cell carcinoma (OSCC) from the surrounding soft and bony tissues with high sensitivity and specificity, optimizing tissue removal and improving patient outcomes [56,66,72,73,74]. Furthermore, Li X et al. published a study on the combination of RS with deep learning algorithms to provide a rapid, non-invasive, and label-free pathological diagnosis of oral cancer and improve the accuracy of the resection margin evaluation [57].

The application of RS to tumor margin delineation has certain limitations because of the infrequency of Raman scattering events, which results in prolonged intraoperative investigation [67]. This limitation led to the development of point spectra via handheld fiber-optic probes, providing diagnostic information at discrete locations [67,75]. Daoust et al. developed a handheld line scanning system with a spatial resolution of 250 μm and spectral resolution of 6 cm^−1^, allowing Raman imaging to be performed over a field-of-view (FOV) of 95 mm^2^ [48]. Aaboubout et al. reported on a prototype instrument employing a fiber-optic needle probe based on the Raman spectra. The instrument is driven into the specimen, from the resection surface towards the tumor, and collects data along the insertion path at each 0.5 mm of depth and determines the distance between the resection margin and the tumor border [2]. The SpectroPen developed by Mohs, a handheld device, can detect both in vivo fluorescence (indocyanine green, ICG) and SERS contrast agents (pegylated colloidal gold) with a tissue penetration depth of 5–10 mm [55]. Another study demonstrated a fluorescence-guided Raman spectroscopic probe tracking system enabling tumor margin delineation with both white light and fluorescence image guidance [76]. The Raman spectroscopy-based objective IOARM device uses the high wavenumber part of the Raman spectrum through a thin fiber-optic needle probe. This probe can be inserted into the specimen and can rapidly determine the distance between the resection surface and the tumor border. Aaboubout Y et al. elaborated a promising margin length prediction and tissue classification model for the quick and accurate assessment of resection margins [53].

A recently published systematic review reported on a large number of biomolecules (e.g., lipids, proteins, DNA, b-carotene, and amino acids, such as phenylalanine, tryptophan, and tyrosine), discriminating cancer from healthy tissue in certain circumstances [77,78]. These biomolecules may provide the opportunity to detect oral cancer at an early stage, reveal malignant transformation or recurrence, or evaluate the resection margin [70,79,80,81]. A further clinical investigation demonstrated that the water concentration from inside the tumor toward the surgical margin shows a negative gradient, even in bone infiltration of the head neck region [66,82]. These findings may provide an objective intraoperative method for the assessment of resection margins.

### 3.2. Mass Spectrometry

Generally, mass spectrometry (MS) can rapidly analyze the molecular composition of tissues and characterize chemical compounds and substances by separating ions by charge and mass. In clinical research and practice, mass spectrometry (MS) is used for biomarker discovery, including proteomics, lipidomics, and metabolomics, to provide a molecular fingerprint for tissues and differentiate healthy tissue from malignant tissue. [16,63,83,84]. The imbalance in tumor suppressing and promoting factors in cancer cells results in changes in the composition of lipids, metabolites, and proteins. By analyzing the molecular profiles of tissues, it is possible to identify positive resection margins and ensure a complete resection. However, preparation and the reliable detection of lipids provide easier feasibility. Most available MS techniques analyze lipid molecules [63]. Clinical studies have revealed discriminatory peaks in the composition of phosphatidic acid (PA), phosphatidylinositol (PI), diacylglycerols (DAGs), and triacylglycerols (TAGs) [16].

*Desorption electrospray ionization* (DESI) is a combination of electrospray (ESI) and desorption ionization (DI) methods, where electrosprayed, charged droplets and ions in the solvent are directed onto the surface to be analyzed, producing gaseous ions of the material on the surface. The gas-phase ions are transferred into the MS, and the mass-to-charge ratios of the ions and their abundance are measured [85,86]. DESI can be applied to resected tissue, frozen sections, and fresh tissue smears, and depending on the type of solvent, different molecules can be analyzed [63,86]. Several clinical studies have demonstrated its enormous potential in the intraoperative assessment of surgical margins in gastric, pancreatic, brain, or breast cancer, showing excellent histological specificity and tissue classification [63,86,87,88,89]. Regarding oral cancer, various clinical trials have shown that DESI can accurately (>90%) determine the mucosal margin of OSCC, although further clinical studies are required to evaluate the deep margin and characteristic lipid molecules to predict prognosis [90,91]. Furthermore, not only does the resection margin state have a diagnostic value, but also saliva containing OSCC metabolite signatures [92]. Remarkable enhancements can be observed in the MS technique, leading to the development of handheld devices and, consequently, the opportunity for fast and easy application in clinical practice.

*Rapid evaporative ionization mass spectrometry* (REIMS) applies standard electrosurgical methods (rapid thermal evaporation) to yield gaseous molecular ions of the tissue components in vivo or ex vivo, so it does not require tissue preparation and uses a spectral library and principal component analysis [93,94]. The aerosol, a rich source of biological information, released during electrosurgical dissection is characterized by REIMS in near real time. The first application, mentioned as iKnife, was described in a previous publication, followed by data collection from gastric, colonic, hepatic, breast, lung, and brain tissue, and the development of a spectral reference library, revealing 100% accuracy [95]. Interestingly, differences were detected between the environments of metastatic and primary tumors (altered membrane lipid composition of histologically healthy cells around the primary tumor), which also supports field cancerization theory [47,95,96,97]. Following the determination of the accuracy of REIMS for intraoperative margin assessment in prospective multicenter clinical studies, it may lead to individualized oncological management of patients [47,59,60,63,98].

The *picosecond infrared laser* (PIRL) can rapidly extract tissue molecular content expanding in the atmosphere in the gas phase via a desorptive mechanism, without significant thermal damage [99]. The capture and analysis, using mass spectrometry, of these gaseous molecular ions are possible with coupling to an appropriate post-desorption ionization source for MS imaging applications [99]. PIRL is feasible not only to analyze phospholipids, but also the protein content of tissues under unaltered conditions, with preserved enzymatic activities [100,101]. Recently, tissue-specific MS profiles were obtained within 5–10 s after tissue ablation with a handheld PIRL device, demonstrating the opportunity for intraoperative use and real-time analysis [63,99,102]. Regarding head and neck surgery, a preliminary study revealed the superiority of PIRL ablation in cutting precision, with less collateral tissue damage to soft and bony tissues. However, further clinical investigations are required [61,103].

The *MasSpec Pen* is a pen-sized handheld device that allows time- and volume-controlled molecular sampling from tissues in vivo and ex vivo, using a discrete water droplet and transporting it to the MS [104]. Several studies have demonstrated its high sensitivity, specificity, and accuracy for the diagnosis of different types of cancer [58,104]. The probe, providing localized molecular information, may facilitate intraoperative use and be a useful tool to guide surgery [62,105].

*SpiderMass*, an MS-based mobile approach, uses water-assisted laser desorption and ionization. This analytical method is feasible to analyze in vivo and in real time the lipido-metabolic molecular profiles on the surface of biological tissues, such as OSCC [16,64,106,107]. The system can be used intra- and postoperatively, and as retrospective analysis in pathology [108]. As well as the handheld probe mentioned above, SpiderMass is also appropriate for precisely defining the resection margin during the excision [107,108].

*Gas chromatography–mass spectrometry (GC/MS)*, a combination of gas chromatography and mass spectrometry, can identify different substances within a wide range of test samples. Yang et al. developed a panel of metabolites based on the GC/MS technique to evaluate negative and dysplastic margins. Their study revealed that specific enzyme activity in dysplastic surgical margins may be a predictor of tumor recurrence for OSCC patients [109].

### 3.3. Further Spectral Imaging Techniques

Fluorescence spectra are collected superficially, which are influenced by the excitation wavelength, investigated oral site (e.g., degree of keratinization), biochemical composition, and tissue architecture. *Fluorescence spectroscopy* is a non-invasive optical visualization method, which involves a beam of light that excites the electrons in molecules and causes them to emit light. This imaging technique has shown certain accuracy in the diagnosis and evaluation of cellular changes and can be supplemented with contrast agents (e.g., indocyanine green) or antibodies (e.g., epidermal growth factor receptor) to increase the accuracy of this modality [110,111,112,113,114]. Near-infrared (NIR) fluorescence imaging facilitates real-time margin assessment and guides surgical resection [113,115]. NIR fluorescence imaging has been used successfully in several research studies and clinical procedures for intraoperative image-guided tumor resection, and improved negative margins were observed [113,116].

*Optical coherence tomography* (OCT) is a high-resolution microstructural imaging technique that also uses NIR light to obtain micrometer-level depth resolution. The transverse scanning of the light beam can produce two- and three-dimensional images from light reflected from within the investigated samples [117,118]. Hamdoon Z et al. investigated OSCC tissue ex vivo and found overall high sensitivity (81.5%), specificity (87%), and accuracy (88%) [117]. OCT can identify architectural changes in the tumor margin, as well as field cancerization [117,118]. The first intraoperative application was published by Sunny SP, and OCT significantly differentiated OSCC from dysplastic lesions or healthy tissue, visualizing the microarchitecture of the resected tissues without any changes in the specimen integrity or clinical workflow [118,119]. Furthermore, the automatic identification algorithm for OCT images based on deep learning may provide decision support for the screening and diagnosis of oral cancer [120].

*Confocal laser endomicroscopy* (CLE) is an endoscopic-assisted, non-invasive imaging technique that can obtain histopathological diagnoses in real time [121]. CLE can also be used in combination with contrast to visualize cellular and architectural characteristics of tissue, with high resolution. The low-intensity laser light emitted by the scanner probe is focused at an adjustable focus depth. CLE allows for the in vivo visualization of cellular and subcellular structures on the epithelial and subepithelial surface of the anterior human oropharyngeal region with high resolution and frame rates, using acriflavine topically and fluorescein intravenously [122]. Several studies have demonstrated promising results, including a scoring system to classify benign and malignant tissue in the oral cavity, and an exceptionally high sensitivity and specificity [123,124,125]. Another study suggested that CLE may supplant or reduce the need for physical tissue biopsy in the management of oral cancer [126]. Horgan CC et al. developed and applied a novel hybrid fiber-optic confocal Raman endomicroscopy system for morpho-chemical tissue imaging and analysis, demonstrating real-time microscopic visualization and simultaneous pointwise label-free biomolecular characterization [127].

*Laser-induced breakdown spectroscopy* (LIBS), an atomic emission spectroscopy, has been studied as a potential method for detecting oral cancer. The excitation source of the technique is a highly energetic laser pulse with a certain threshold for optical breakdown focusing to form a plasma, which atomizes and excites samples depending on the environment and the target materials. The method provides direct measurement with real-time examination of a minimal tissue sample, and it can distinguish tumorous and healthy tissue. Winnand P et al. investigated the microscopic tumor spread of oral cancer in bone with LIBS and found that this method may provide a possibility to define the resection margin status in bone-invasive oral cancer, which is a quintessential problem in oncologic surgery because of the lack of rapid bone analysis methods [128]. Winnand P et al. demonstrated robust real-time detection of bone involvement with LIBS. However, further studies are required to evaluate its applicability and safety during oncological surgery [128].

## 4. Artificial Intelligence and Spectroscopy in the Intraoperative Assessment of Tumor Resection

Radiological or histological imaging play an essential role in the diagnosis, staging, and further management of oral cancer. However, as mentioned above, spectroscopic imaging methods have the potential to contribute to oncological management [129]. One of the greatest achievements of the 21st century is artificial intelligence (AI) and its involvement in the analysis of diagnostics data. There have been numerous studies published, where AI has been utilized in interpreting spectroscopic data. These methods have demonstrated high accuracy in identifying malignant lesions, while requiring minimal sample preparation and short working time. By leveraging AI algorithms, spectroscopic methods can provide clinicians with valuable insights into the cellular and subcellular structures and functions associated with cancerous tissues. These findings suggest the possibility of influencing the decision-making process in real time with the ultimate aim of improving oncological patient care. The integration of spectroscopic imaging methods with AI has the potential to revolutionize the field of oncological management, particularly in the context of oral cancer [130,131,132,133,134].

## 5. Conclusion and Future Directions

Surgical excision will remain the gold standard method for tumors in the head and neck region, and an adequate resection margin is the key to survival and the local recurrence rate. Although FS is a widely used method to determine the resection margin, it is also a time-consuming and resource-intensive procedure that cannot guide the surgeon in the real-time assessment of tissue resection. However, the emergence of real-time visualization spectroscopic techniques in the operating room is innovative and foreshadows significant progress. These spectroscopic techniques have the potential to guide the surgery and assist in determining the tumor-free resection margin. Although several different spectroscopic approaches are currently available; unfortunately, they have been studied with inhomogeneous methodologies. There is currently no available publication comparing the efficacy of these methods, or the specificity of the investigated parameters, in oral cancer. Therefore, long-term prospective, multicentric clinical studies are still needed to standardize the intraoperative assessment of the resection margin and establish the optimal use of spectroscopic approaches. The combination of spectroscopic imaging methods and AI may represent a significant advancement in oncological management, specifically for oral cancer. The integration of these technologies has the potential to enhance diagnostic accuracy, streamline treatment planning, and ultimately improve the care and survival of patients. Continued research and development are vital to fully harness the power of this novel approach and integrate it effectively into clinical practice.

## Figures and Tables

**Table 1:** Risk factors of oral cancer.

Modifiable Risk Factors	Non-Modifiable Risk Factors
Smoking and smokeless tobacco (e.g., betel quid or snuff) Alcohol Sun exposure Poor oral hygiene Chronic oral inflammation	Gender Age Pre-/malignant lesions (e.g., leukoplakia) Immunosuppressive conditions and genetic comorbidities (e.g., HIV, Plummer–Vinson syndrome, Li–Fraumeni syndrome, Fanconi anemia, dyskeratosis congenita) Human papillomavirus

**Table 2:** Prognostic factors impacting the 5-year survival rate in oral cancer.

Main Factors	Further Influencing Factors
Age Tumor TNM stage, sites Histological grading	Time between disease and perception Related treatment Access to healthcare services Educational level and occupation of the patient Behavioral/cultural factors Exposure to modifiable and non-modifiable risk factors

**Table 3:** Currently available intraoperative spectrometric devices for head and neck oncological surgery.

Methods and Devices		Examined Tissues	Tested Ex Vivo or In Vivo	Diagnostic or Surgical Tools
Raman spectroscopy	Line scanning system [48]	Porcine tissue	Ex vivo	Diagnostic
	Fiber-optic needle probe [48,54]	Human tongue, mandible	Ex vivo	Diagnostic
	SpectroPen [55,56,57]	Murine mammary cancer tissue, human skin squamous cell carcinoma	Ex vivo and in vivo	Diagnostic
Mass spectroscopy	DESI [58]	Human gastric, pancreatic, brain, breast cancer, and oral cancer	Ex vivo	Diagnostic
	iKnife [47,59,60]	Human brain, colorectal, breast, gastric, colonic, hepatic, and lung	In vivo	Diagnostic and surgical
	PIRL [61]	Porcine laryngeal tissue, human brain, and breast cancer	Ex vivo	Diagnostic and potentially surgical
	MasSpec Pen [58,62,63]	Human thyroid, parathyroid, lymph node, breast, pancreatic, and bile duct malignant tissues	Ex vivo	Diagnostic
	SpiderMass [16,64]	Dog sarcoma tissue	Ex vivo and in vivo	Diagnostic

## References

[1] Ferlay J., Colombet M., Soerjomataram I., Mathers C., Parkin D.M., Piñeros M., Znaor A., Bray F. (2019). Estimating the global cancer incidence and mortality in 2018: GLOBOCAN sources and methods. Int. J. Cancer.

[2] Aaboubout Y., Ten Hove I., Smits R.W.H., Hardillo J.A., Puppels G.J., Koljenovic S. (2021). Specimen-driven intraoperative assessment of resection margins should be standard of care for oral cancer patients. Oral Dis..

[3] Blot W.J., McLaughlin J.K., Winn D.M., Austin D.F., Greenberg R.S., Preston-Martin S., Bernstein L., Schoenberg J.B., Stemhagen A., Fraumeni J.F. (1988). Smoking and drinking in relation to oral and pharyngeal cancer. Cancer Res..

[4] Hashibe M., Brennan P., Chuang S.C., Boccia S., Castellsague X., Chen C., Curado M.P., Dal Maso L., Daudt A.W., Fabianova E. (2009). Interaction between tobacco and alcohol use and the risk of head and neck cancer: Pooled analysis in the International Head and Neck Cancer Epidemiology Consortium. Cancer Epidemiol. Biomarkers Prev..

[5] Rettig E.M., D’Souza G. (2015). Epidemiology of head and neck cancer. Surg. Oncol. Clin. N. Am..

[6] Kumar M., Nanavati R., Modi T.G., Dobariya C. (2016). Oral cancer: Etiology and risk factors: A review. J. Cancer Res. Ther..

[7] Vokes E.E., Agrawal N., Seiwert T.Y. (2015). HPV-Associated Head and Neck Cancer. J. Natl. Cancer Inst..

[8] Auperin A. (2020). Epidemiology of head and neck cancers: An update. Curr. Opin. Oncol..

[9] Pulte D., Brenner H. (2010). Changes in survival in head and neck cancers in the late 20th and early 21st century: A period analysis. Oncologist.

[10] William M. (2017). Head and Neck Cancers-Major Changes in the American Joint Committee on Cancer Eighth Edition Cancer Staging Manual. CA Cancer J. Clin..

[11] National Cancer Institute, Surveillance, Epidemiology, and End Results (SEER) Program. https://seer.cancer.gov/archive/csr/1975_2018/.

[12] Ebrahimi A., Gil Z., Amit M., Yen T.C., Liao C.T., Chaturvedi P., Agarwal J.P., Kowalski L.P., Kreppel M., International Consortium for Outcome Research (ICOR) in Head and Neck Cancer (2014). Primary tumor staging for oral cancer and a proposed modification incorporating depth of invasion: An international multicenter retrospective study. JAMA Otolaryngol. Head Neck Surg..

[13] Ooms M., Ponke L., Winnand P., Heitzer M., Peters F., Steiner T., Hölzle F., Modabber A. (2023). Predictive factors and repetition numbers for intraoperative additional resection of initially involved soft tissue resection margins in oral squamous cellcarcinoma: A retrospective study. World J. Surg. Oncol..

[14] Chaturvedi P., Datta S., Nair S., Nair D., Pawar P., Vaishampayan S., Patil A., Kane S. (2014). Gross examination by the surgeon as an alternative to frozen section for assessment of adequacy of surgical margin in head and neck squamous cell carcinoma. Head Neck.

[15] Smits R.W., Koljenović S., Hardillo J.A., Ten Hove I., Meeuwis C.A., Sewnaik A., Dronkers E.A., Bakker Schut T.C., Langeveld T.P., Molenaar J. (2016). Resection margins in oral cancer surgery: Room for improvement. Head Neck.

[16] Ogrinc N., Attencourt C., Colin E., Boudahi A., Tebbakha R., Salzet M., Testelin S., Dakpé S., Fournier I. (2022). Mass Spectrometry-Based Differentiation of Oral Tongue Squamous Cell Carcinoma and Nontumor Regions with the SpiderMass Technology. Front. Oral Health.

[17] Nason R.W., Binahmed A., Pathak K.A., Abdoh A.A., Sándor G.K. (2009). What is the adequate margin of surgical resection in oral cancer?. Oral Surg. Oral Med. Oral Pathol. Oral Radiol. Endod..

[18] Helliwell T., Woolgar J. (2013). Standards and Datasets for Reporting Cancers. Dataset for Histopathology Reporting of Mucosal Malignancies of the Oral Cavity. London: Royal College of Pathologists. https://www.rcpath.org/static/6201bef5-79df-4107-ba6a42833377457f/g111_pharynxmucosaldataset_nov13.pdf.

[19] Hinni M.L., Ferlito A., Brandwein-Gensler M.S., Takes R.P., Silver C.E., Westra W.H., Seethala R.R., Rodrigo J.P., Corry J., Bradford C.R. (2013). Surgical margins in head and neck cancer: A contemporary review. Head Neck.

[20] Lin A. (2018). Radiation Therapy for Oral Cavity and Oropharyngeal Cancers. Dent. Clin. N. Am..

[21] Rubin H. (2011). Fields and field cancerization: The preneoplastic origins of cancer: Asymptomatic hyperplastic fields are precursors of neoplasia, and their progression to tumors can be tracked by saturation density in culture. Bioessays.

[22] Slaughter D.P., Southwick H.W., Smejkal W. (1953). Field cancerization in oral stratified squamous epithelium; clinical implications of multicentric origin. Cancer.

[23] Dakubo G.D., Jakupciak J.P., Birch-Machin M.A., Parr R.L. (2007). Clinical implications and utility of field cancerization. Cancer Cell Int..

[24] Goerres G.W., Schmid D.T., Schuknecht B., Eyrich G.K. (2005). Bone invasion in patients with oral cavity cancer: Comparison of conventional CT with PET/CT and SPECT/CT. Radiology.

[25] Van Cann E.M., Koole R., Oyen W.J., de Rooy J.W., de Wilde P.C., Slootweg P.J., Schipper M., Merkx M.A., Stoelinga P.J. (2008). Assessment of mandibular invasion of squamous cell carcinoma by various modes of imaging: Constructing a diagnostic algorithm. Int. J. Oral Maxillofac. Surg..

[26] Wilson L.B. (1905). A method for the rapid preparation of fresh tissues for the microscope. J. Am. Med. Assoc..

[27] Buchakjian M.R., Ginader T., Tasche K.K., Pagedar N.A., Smith B.J., Sperry S.M. (2018). Independent Predictors of Prognosis Based on Oral Cavity Squamous Cell Carcinoma Surgical Margins. Otolaryngol. Head Neck Surg..

[28] Datta S., Mishra A., Chaturvedi P., Bal M., Nair D., More Y., Ingole P., Sawakare S., Agarwal J.P., Kane S.V. (2019). Frozen section is not cost beneficial for the assessment of margins in oral cancer. Indian J. Cancer.

[29] Namdar Z.M., Omidifar N., Arasteh P., Akrami M., Tahmasebi S., Nobandegani A.S., Sedighi S., Zangouri V., Talei A. (2021). How accurate is frozen section pathology compared to permanent pathology in detecting involved margins and lymph nodes in breast cancer?. World J. Surg. Oncol..

[30] Maxwell J.H., Thompson L.D., Brandwein-Gensler M.S., Weiss B.G., Canis M., Purgina B., Prabhu A.V., Lai C., Shuai Y., Carroll W.R. (2015). Early Oral Tongue Squamous Cell Carcinoma: Sampling of Margins from Tumor Bed and Worse Local Control. JAMA Otolaryngol. Head Neck Surg..

[31] Varvares M.A., Poti S., Kenyon B., Christopher K., Walker R.J. (2015). Surgical margins and primary site resection in achieving local control in oral cancer resections. Laryngoscope.

[32] Amit M., Na’ara S., Leider-Trejo L., Akrish S., Cohen J.T., Billan S., Gil Z. (2016). Improving the rate of negative margins after surgery for oral cavity squamous cell carcinoma: A prospective randomized controlled study. Head Neck.

[33] Kain J.J., Birkeland A.C., Udayakumar N., Morlandt A.B., Stevens T.M., Carroll W.R., Rosenthal E.L., Warram J.M. (2020). Surgical margins in oral cavity squamous cell carcinoma: Current practices and future directions. Laryngoscope.

[34] Kubik M.W., Sridharan S., Varvares M.A., Zandberg D.P., Skinner H.D., Seethala R.R., Chiosea S.I. (2020). Intraoperative Margin Assessment in Head and Neck Cancer: A Case of Misuse and Abuse?. Head Neck Pathol..

[35] Maharaj D.D., Thaduri A., Jat B., Poonia D.R., Durgapal P., Rajkumar K.S. (2021). Performance and survival outcomes of defect-driven versus specimen-drivenmethod of frozen section intraoperative margin assessment in oral cancers. Int. J. Oral Maxillofac. Surg..

[36] Smits R.W.H., van Lanschot C.G.F., Aaboubout Y., de Ridder M., Hegt V.N., Barroso E.M., Meeuwis C.A., Sewnaik A., Hardillo J.A., Monserez D. (2020). Intraoperative Assessment of the Resection Specimen Facilitates Achievement of Adequate Margins in Oral Carcinoma. Front. Oncol..

[37] Aaboubout Y., Barroso E.M., Algoe M., Ewing-Graham P.C., ten Hove I., Mast H., Hardillo J.A., Sewnaik A., Monserez D.A., Keereweer S. (2021). Intraoperative Assessment of Resection Margins in Oral Cavity Cancer: This is the Way. J. Vis. Exp..

[38] Weijers M., Snow G.B., Bezemer D.P., van dr Wal J.E., van der Waal I. (2004). The status of the deep surgical margins in tongue and floor of mouth squamous cell carcinoma and risk of local recurrence; an analysis of 68 patients. Int. J. Oral Maxillofac. Surg..

[39] Woolgar J.A., Triantafyllou A. (2005). A histopathological appraisal of surgical margins in oral and oropharyngeal cancer resection specimens. Oral Oncol..

[40] Brouwer de Koning S.G., Schaeffers A.W.M.A., Schats W., van den Brekel M.W.M., Ruers T.J.M., Karakullukcu M.B. (2021). Assessment of the deep resection margin during oral cancer surgery: A systematic review. Eur. J. Surg. Oncol..

[41] Ridha H., Garioch J.J., Tan E.K., Heaton M.J., Igali L., Moncrieff M.D. (2015). Intraoperative use of Mohs’ surgery for the resection of major cutaneous head and neck cancer under general anaesthetic: Initial experiences, efficiency and outcomes. J. Plast. Reconstr. Aesthet. Surg..

[42] Mahmood S., Conway D., Ramesar K.C. (2001). Use of intraoperative cytologic assessment of mandibular marrow scrapings to predict resection margin status in patients with squamous cell carcinoma. J. Oral Maxillofac. Surg..

[43] Nieberler M., Häußler P., Kesting M.R., Kolk A., Deppe H., Weirich G., Wolff K.D. (2016). Clinical Impact of Intraoperative Cytological Assessment of Bone Resection Margins in Patients with Head and Neck Carcinoma. Ann. Surg. Oncol..

[44] Zeng B., Yang L., Liang Y.J., Lao X.M., Mei X.Y., Liao G.Q. (2020). Diagnostic value of intraoperative bone marrow assessment for bone marginsin patients with head and neck squamous cell carcinoma: A systematic review and meta-analysis. Int. J. Oral Maxillofac. Surg..

[45] Clark D.J., Mao L. (2017). Understanding the Surgical Margin: A Molecular Assessment. Oral Maxillofac. Surg. Clin. N. Am..

[46] Puppels G.J., Barroso E.M.L., Aaboubout Y., Nunes Soares M.R., Artyushenko V.G., Bocharnikov A. Intra-operative assessment of tumor resection margins by Raman spectroscopy to guide oral cancer surgery (Conference Presentation). Proceedings of the Biomedical Vibrational Spectroscopy 2020: Advances in Research and Industry.

[47] Tzafetas M., Mitra A., Paraskevaidi M., Bodai Z., Kalliala I., Bowden S., Lathouras K., Rosini F., Szasz M., Savage A. (2020). The intelligent knife (iKnife) and its intraoperative diagnostic advantage for the treatment of cervical disease. Proc. Natl. Acad. Sci. USA.

[48] Daoust F., Tavera H., Dallaire F., Orsini P., Savard K., Bismuth J., Mckoy P., Veilleux I., Petrecca K., Leblond F. (2023). A clinical Raman spectroscopy imaging system and safety requirements for in situ intraoperative tissue characterization. Analyst.

[49] Raman C.V., Krishnan K.S. (1928). A New Type of Secondary Radiation. Nature.

[50] Thomas N.C. (1991). The early history of spectroscopy. J. Chem. Educ..

[51] Dodo K., Fujita K., Sodeoka M. (2022). Raman Spectroscopy for Chemical Biology Research. J. Am. Chem. Soc..

[52] Ren J., Mao S., Lin J., Xu Y., Zhu Q., Xu N. (2022). Research Progress of Raman Spectroscopy and Raman Imaging in Pharmaceutical Analysis. Curr. Pharm. Des..

[53] Aaboubout Y., Nunes Soares M.R., Bakker Schut T.C., Barroso E.M., van der Wolf M., Sokolova E., Artyushenko V., Bocharnikov A., Usenov I., van Lanschot C.G.F. (2023). Intraoperative assessment of resection margins by Raman spectroscopy to guide oral cancer surgery. Analyst.

[54] Shawn P., Mulvaney, Christine D.K. (2000). Raman Spectroscopy. Anal. Chem..

[55] Mohs A.M., Mancini M.C., Singhal S., Provenzale J.M., Leyland-Jones B., Wang M.D., Nie S. (2010). Hand-held spectroscopic device for in vivo and intraoperative tumor detection: Contrast enhancement, detection sensitivity, and tissue penetration. Anal. Chem..

[56] Holler S., Haig B., Donovan M.J., Sobrero M., Miles B.A. (2018). A monolithic microsphere-fiber probe for spatially resolved Raman spectroscopy: Application to head and neck squamous cell carcinomas. Rev. Sci. Instrum..

[57] Li X., Li L., Sun Q., Chen B., Zhao C., Dong Y., Zhu Z., Zhao R., Ma X., Yu M. (2023). Rapid multi-task diagnosis of oral cancer leveraging fiber-optic Ramanspectroscopy and deep learning algorithms. Front. Oncol..

[58] Brown H.M., Pirro V., Cooks R.G. (2018). From DESI to the MasSpec Pen: Ambient Ionization Mass Spectrometry for Tissue Analysis and Intrasurgical Cancer Diagnosis. Clin. Chem..

[59] St John E.R., Balog J., McKenzie J.S., Rossi M., Covington A., Muirhead L., Bodai Z., Rosini F., Speller A.V.M., Shousha S. (2017). Rapid evaporative ionisation mass spectrometry of electrosurgical vapours for the identification of breast pathology: Towards an intelligent knife for breast cancer surgery. Breast Cancer Res..

[60] Phelps D.L., Balog J., Gildea L.F., Bodai Z., Savage A., El-Bahrawy M.A., Speller A.V., Rosini F., Kudo H., McKenzie J.S. (2018). The surgical intelligent knife distinguishes normal, borderline and malignant gynaecological tissues using rapid evaporative ionisation mass spectrometry (REIMS). Br. J. Cancer.

[61] Jowett N., Wöllmer W., Reimer R., Zustin J., Schumacher U., Wiseman P.W., Mlynarek A.M., Böttcher A., Dalchow C.V., Lörincz B.B. (2014). Bone ablation without thermal or acoustic mechanical injury via a novel picosecond infrared laser (PIRL). Otolaryngol. Head Neck Surg..

[62] King M.E., Zhang J., Lin J.Q., Garza K.Y., DeHoog R.J., Feider C.L., Bensussan A., Sans M., Krieger A., Badal S. (2021). Rapid diagnosis and tumor margin assessment during pancreatic cancer surgery with the MasSpec Pen technology. Proc. Natl. Acad. Sci. USA.

[63] Hänel L., Kwiatkowski M., Heikaus L., Schlüter H. (2019). Mass spectrometry-based intraoperative tumor diagnostics. Future Sci. OA.

[64] Ogrinc N., Saudemont P., Balog J., Robin Y.M., Gimeno J.P., Pascal Q., Tierny D., Takats Z., Salzet M., Fournier I. (2019). Water-assisted laser desorption/ionization mass spectrometry for minimally invasive in vivo and real-time surface analysis using SpiderMass. Nat. Protoc..

[65] Gardiner D.J. (1989). Practical Raman Spectroscopy.

[66] Barroso E.M., Ten Hove I., Bakker Schut T.C., Mast H., van Lanschot C.G.F., Smits R.W.H., Caspers P.J., Verdijk R., Noordhoek Hegt V., de Jong R.J.B. (2018). Raman spectroscopy for assessment of bone resection margins in mandibulectomy for oral cavity squamous cell carcinoma. Eur. J. Cancer.

[67] Pence I., Mahadevan-Jansen A. (2016). Clinical instrumentation and applications of Raman spectroscopy. Chem. Soc. Rev..

[68] Jones R.R., Hooper D.C., Zhang L., Wolverson D., Valev V.K. (2019). Raman Techniques: Fundamentals and Frontiers. Nanoscale Res. Lett..

[69] Koljenović S., Schut T.B., Vincent A., Kros J.M., Puppels G.J. (2005). Detection of meningioma in dura mater by Raman spectroscopy. Anal. Chem..

[70] Haka A.S., Volynskaya Z., Gardecki J.A., Nazemi J., Lyons J., Hicks D., Fitzmaurice M., Dasari R.R., Crowe J.P., Feld M.S. (2006). In vivo margin assessment during partial mastectomy breast surgery using Raman spectroscopy. Cancer Res..

[71] Bergholt M.S., Lin K., Wang J., Zheng W., Xu H., Huang Q., Ren J.L., Ho K.Y., Teh M., Srivastava S. (2016). Simultaneous fingerprint and high-wavenumber fiber-optic Raman spectroscopy enhances real-time in vivo diagnosis of adenomatous polyps during colonoscopy. J. Biophotonics.

[72] Gregory W.A.S., Kiran K., Changhe H., Brandy B., Micaela T., Zachary A., Angela E., Katlyn C.M., Michelle A.B. (2018). Applications of Raman spectroscopy in cancer diagnosis. Cancer Metastasis Rev..

[73] Barroso E.M., Smits R.W., Bakker S.T.C., ten Hove I., Hardillo J.A., Wolvius E.B., Baatenburg de Jong R.J., Koljenović S., Puppels G.J. (2015). Discrimination between oral cancer and healthy tissue based on water content determined by Raman spectroscopy. Anal. Chem..

[74] Christian K., Johanna M., Werner A., Kathrin B., Tesfay G.M., Robert H., Abbas A., Stefan W., Andreas B., Wilhelm N.F. (2014). Raman difference spectroscopy: A non-invasive method for identification of oral squamous cell carcinoma. Biomed. Opt. Express.

[75] Puppels G.J., de Mul F.F., Otto C., Greve J., Robert-Nicoud M., Arndt-Jovin D.J., Jovin T.M. (1990). Studying single living cells and chromosomes by confocal Raman microspectroscopy. Nature.

[76] Horgan C.C., Bergholt M.S., Thin M.Z., Nagelkerke A., Kennedy R., Kalber T.L., Stuckey D.J., Stevens M.M. (2021). Image-guided Raman spectroscopy probe-tracking for tumor margin delineation. J. Biomed. Opt..

[77] Thomas Robbins K., Triantafyllou A., Suárez C., López F., Hunt J.L., Strojan P., Williams M.D., Braakhuis B.J.M., de Bree R., Hinni M.L. (2019). Surgical margins in head and neck cancer: Intra- and postoperative considerations. Auris Nasus Larynx.

[78] Faur C.I., Falamas A., Chirila M., Roman R.C., Rotaru H., Moldovan M.A., Albu S., Baciut M., Robu I., Hedesiu M. (2022). Raman spectroscopy in oral cavity and oropharyngeal cancer: A systematic review. Int. J. Oral Maxillofac. Surg..

[79] Sahu A., Sawant S., Mamgain H., Krishna C.M. (2013). Raman spectroscopy of serum: An exploratory study for detection of oral cancers. Analyst.

[80] Knipfer C., Motz J., Adler W., Brunner K., Gebrekidan M.T., Hankel R., Agaimy A., Will S., Braeuer A., Neukam F.W. (2015). Raman difference spectroscopy: A non-invasive method for identification of oral squamous cell carcinoma: Publisher’s note. Biomed. Opt. Express.

[81] Xue L., Yan B., Li Y., Tan Y., Luo X., Wang M. (2018). Surface-enhanced Raman spectroscopy of blood serum based on gold nanoparticles for tumor stages detection and histologic grades classification of oral squamous cell carcinoma. Int. J. Nanomed..

[82] Barroso E.M., Smits R.W., van Lanschot C.G., Caspers P.J., Ten Hove I., Mast H., Sewnaik A., Hardillo J.A., Meeuwis C.A., Verdijk R. (2016). Water Concentration Analysis by Raman Spectroscopy to Determine the Location of the Tumor Border in Oral Cancer Surgery. Cancer Res..

[83] Zhou W., Petricoin E.F., Longo C. (2017). Mass Spectrometry-Based Biomarker Discovery. Methods Mol. Biol..

[84] Santilli A.M.L., Ren K., Oleschuk R., Kaufmann M., Rudan J., Fichtinger G., Mousavi P. (2022). Application of Intraoperative Mass Spectrometry and Data Analytics for Oncological Margin Detection, A Review. IEEE Trans. Biomed. Eng..

[85] Takats Z., Wiseman J.M., Gologan B., Cooks R.G. (2004). Mass spectrometry sampling under ambient conditions with desorption electrospray ionization. Science.

[86] Cooks R.G., Ouyang Z., Takats Z., Wiseman J.M. (2006). Detection technologies. ambient mass spectrometry. Science.

[87] Calligaris D., Caragacianu D., Liu X., Norton I., Thompson C.J., Richardson A.L., Golshan M., Easterling M.L., Santagata S., Dillon D.A. (2014). Application of desorption electrospray ionization mass spectrometry imaging in breast cancer margin analysis. Proc. Natl. Acad. Sci. USA.

[88] Eberlin L.S., Margulis K., Planell-Mendez I., Zare R.N., Tibshirani R., Longacre T.A., Jalali M., Norton J.A., Poultsides G.A. (2016). Pancreatic Cancer Surgical Resection Margins: Molecular Assessment by Mass Spectrometry Imaging. PLoS Med..

[89] Pirro V., Alfaro C.M., Jarmusch A.K., Hattab E.M., Cohen-Gadol A.A., Cooks R.G. (2017). Intraoperative assessment of tumor margins during glioma resection by desorption electrospray ionization-mass spectrometry. Proc. Natl. Acad. Sci. USA.

[90] D’Hue C., Moore M., Summerlin D.J., Jarmusch A., Alfaro C., Mantravadi A., Bewley A., Gregory F.D., Cooks R.G. (2018). Feasibility of desorption electrospray ionization mass spectrometry for diagnosis of oral tongue squamous cell carcinoma. Rapid Commun. Mass. Spectrom..

[91] Yang X., Song X., Zhang X., Shankar V., Wang S., Yang Y., Chen S., Zhang L., Ni Y., Zare R.N. (2021). In situ DESI-MSI lipidomic profiles of mucosal margin of oral squamous cell carcinoma. EBioMedicine.

[92] Song X., Yang X., Narayanan R., Shankar V., Ethiraj S., Wang X., Duan N., Ni Y.H., Hu Q., Zare R.N. (2020). Oral squamous cell carcinoma diagnosed from saliva metabolic profiling. Proc. Natl. Acad. Sci. USA.

[93] Schäfer K.C., Dénes J., Albrecht K., Szaniszló T., Balog J., Skoumal R., Katona M., Tóth M., Balogh L., Takáts Z. (2009). In vivo, in situ tissue analysis using rapid evaporative ionization mass spectrometry. Angew. Chem. Int. Ed. Engl..

[94] Jones E.A., Simon D., Karancsi T., Balog J., Pringle S.D., Takats Z. (2019). Matrix Assisted Rapid Evaporative Ionization Mass Spectrometry. Anal. Chem..

[95] Balog J., Sasi-Szabó L., Kinross J., Lewis M.R., Muirhead L.J., Veselkov K., Mirnezami R., Dezső B., Damjanovich L., Darzi A. (2013). Intraoperative tissue identification using rapid evaporative ionization mass spectrometry. Sci. Transl. Med..

[96] Yakoub D., Keun H.C., Goldin R., Hanna G.B. (2010). Metabolic profiling detects field effects in nondysplastic tissue from esophageal cancer patients. Cancer Res..

[97] Cacho-Díaz B., García-Botello D.R., Wegman-Ostrosky T., Reyes-Soto G., Ortiz-Sánchez E., Herrera-Montalvo L.A. (2020). Tumor microenvironment differences between primary tumor and brain metastases. J. Transl. Med..

[98] Janssen N.N.Y., Kaufmann M., Santilli A., Jamzad A., Vanderbeck K., Ren K.Y.M., Ungi T., Mousavi P., Rudan J.F., McKay D. (2020). Navigated tissue characterization during skin cancer surgery. Int. J. Comput. Assist. Radiol. Surg..

[99] Woolman M., Ferry I., Kuzan-Fischer C.M., Wu M., Zou J., Kiyota T., Isik S., Dara D., Aman A., Das S. (2017). Rapid determination of medulloblastoma subgroup affiliation with mass spectrometry using a handheld picosecond infrared laser desorption probe. Chem. Sci..

[100] Kwiatkowski M., Wurlitzer M., Omidi M., Ren L., Kruber S., Nimer R., Robertson W.D., Horst A., Miller R.J., Schlüter H. (2015). Ultrafast extraction of proteins from tissues using desorption by impulsive vibrational excitation. Angew. Chem. Int. Ed. Engl..

[101] Zou J., Talbot F., Tata A., Ermini L., Franjic K., Ventura M., Zheng J., Ginsberg H., Post M., Ifa D.R. (2015). Ambient Mass Spectrometry Imaging with Picosecond Infrared Laser Ablation Electrospray Ionization (PIR-LAESI). Anal. Chem..

[102] Woolman M., Kuzan-Fischer C.M., Ferry I., Kiyota T., Luu B., Wu M., Munoz D.G., Das S., Aman A., Taylor M.D. (2019). Picosecond Infrared Laser Desorption Mass Spectrometry Identifies Medulloblastoma Subgroups on Intrasurgical Timescales. Cancer Res..

[103] Böttcher A., Clauditz T.S., Knecht R., Kucher S., Wöllmer W., Wilczak W., Krötz P., Jowett N., Dalchow C.V., Münscher A. (2013). A novel tool in laryngeal surgery: Preliminary results of the picosecond infrared laser. Laryngoscope.

[104] Zhang J., Rector J., Lin J.Q., Young J.H., Sans M., Katta N., Giese N., Yu W., Nagi C., Suliburk J. (2017). Nondestructive tissue analysis for ex vivo and in vivo cancer diagnosis using a handheld mass spectrometry system. Sci. Transl. Med..

[105] Zhang J., Sans M., DeHoog R.J., Garza K.Y., King M.E., Feider C.L., Bensussan A., Keating M.F., Lin J.Q., Povilaitis S.C. (2021). Clinical Translation and Evaluation of a Handheld and Biocompatible Mass Spectrometry Probe for Surgical Use. Clin. Chem..

[106] Fatou B., Saudemont P., Leblanc E., Vinatier D., Mesdag V., Wisztorski M., Focsa C., Salzet M., Ziskind M., Fournier I. (2016). In vivo Real-Time Mass Spectrometry for Guided Surgery Application. Sci. Rep..

[107] Saudemont P., Quanico J., Robin Y.M., Baud A., Balog J., Fatou B., Tierny D., Pascal Q., Minier K., Pottier M. (2018). Real-Time Molecular Diagnosis of Tumors Using Water-Assisted Laser Desorption/Ionization Mass Spectrometry Technology. Cancer Cell.

[108] Ogrinc N., Caux P.D., Robin Y.M., Bouchaert E., Fatou B., Ziskind M., Focsa C., Bertin D., Tierny D., Takats Z. (2021). Direct Water-Assisted Laser Desorption/Ionization Mass Spectrometry Lipidomic Analysis and Classification of Formalin-Fixed Paraffin-Embedded Sarcoma Tissues without Dewaxing. Clin. Chem..

[109] Yang X.H., Zhang X.X., Jing Y., Ding L., Fu Y., Wang S., Hu S.Q., Zhang L., Huang X.F., Ni Y.H. (2019). Amino acids signatures of distance-related surgical margins of oral squamous cell carcinoma. EBioMedicine.

[110] Grandis J.R., Tweardy D.J. (1993). Elevated levels of transforming growth factor alpha and epidermal growth factor receptor messenger RNA are early markers of carcinogenesis in head and neck cancer. Cancer Res..

[111] Francisco A.L., Correr W.R., Pinto C.A., Gonçalves Filho J., Chulam T.C., Kurachi C., Kowalski L.P. (2014). Analysis of surgical margins in oral cancer using in situ fluorescence spectroscopy. Oral Oncol..

[112] Holt D., Singhal S., Selmic L.E. (2020). Near-infrared imaging and optical coherence tomography for intraoperative visualization of tumors. Vet. Surg..

[113] Stepan K.O., Li M.M., Kang S.Y., Puram S.V. (2020). Molecular margins in head and neck cancer: Current techniques and future directions. Oral Oncol..

[114] Voskuil F.J., de Jongh S.J., Hooghiemstra W.T.R., Linssen M.D., Steinkamp P.J., de Visscher S.A.H.J., Schepman K.P., Elias S.G., Meersma G.J., Jonker P.K.C. (2020). Fluorescence-guided imaging for resection margin evaluation in head and neck cancer patients using cetuximab-800CW: A quantitative dose-escalation study. Theranostics.

[115] Pan J., Deng H., Hu S., Xia C., Chen Y., Wang J., Wang Y. (2020). Real-time surveillance of surgical margins via ICG-based near-infrared fluorescence imaging in patients with OSCC. World J. Surg. Oncol..

[116] Sun X., Chintakunta P.K., Badachhape A.A., Bhavane R., Lee H.J., Yang D.S., Starosolski Z., Ghaghada K.B., Vekilov P.G., Annapragada A.V. (2023). Rational Design of a Self-Assembling High Performance Organic Nanofluorophore for Intraoperative NIR-II Image-Guided Tumor Resection of Oral Cancer. Adv. Sci..

[117] Hamdoon Z., Jerjes W., McKenzie G., Jay A., Hopper C. (2016). Optical coherence tomography in the assessment of oral squamous cell carcinoma resection margins. Photodiagnosis Photodyn. Ther..

[118] Sunny S.P., Agarwal S., James B.L., Heidari E., Muralidharan A., Yadav V., Pillai V., Shetty V., Chen Z., Hedne N. (2019). Intra-operative point-of-procedure delineation of oral cancer margins using optical coherence tomography. Oral Oncol..

[119] Badhey A.K., Schwarz J.S., Laitman B.M., Veremis B.M., Westra W.H., Yao M., Teng M.S., Genden E.M., Miles B.A. (2023). Intraoperative Use of Wide-Field Optical Coherence Tomography to Evaluate Tissue Microstructure in the Oral Cavity and Oropharynx. JAMA Otolaryngol. Head Neck Surg..

[120] Yang Z., Pan H., Shang J., Zhang J., Liang Y. (2023). Deep-Learning-Based Automated Identification and Visualization of Oral Cancer in Optical Coherence Tomography Images. Biomedicines.

[121] Fugazza A., Gaiani F., Carra M.C., Brunetti F., Lévy M., Sobhani I., Azoulay D., Catena F., de’Angelis G.L., de’Angelis N. (2016). Confocal Laser Endomicroscopy in Gastrointestinal and Pancreatobiliary Diseases: A Systematic Review and Meta-Analysis. Biomed. Res. Int..

[122] Haxel B.R., Goetz M., Kiesslich R., Gosepath J. (2010). Confocal endomicroscopy: A novel application for imaging of oral and oropharyngeal mucosa in human. Eur. Arch. Otorhinolaryngol..

[123] Sethi S., Ju X., Logan R.M., Sambrook P., McLaughlin R.A., Jamieson L.M. (2021). Diagnostic Accuracy of Confocal Laser Endomicroscopy for the Diagnosis of Oral Squamous Cell Carcinoma: A Systematic Review and Meta-Analysis. Int. J. Environ. Res. Public. Health.

[124] Sievert M., Oetter N., Mantsopoulos K., Gostian A.O., Mueller S.K., Koch M., Balk M., Thimsen V., Stelzle F., Eckstein M. (2022). Systematic classification of confocal laser endomicroscopy for the diagnosis of oral cavity carcinoma. Oral Oncol..

[125] Villard A., Breuskin I., Casiraghi O., Asmandar S., Laplace-Builhe C., Abbaci M., Moya Plana A. (2022). Confocal laser endomicroscopy and confocal microscopy for head and neck cancer imaging: Recent updates and future perspectives. Oral Oncol..

[126] Farah C.S., Janik M., Woo S.B., Grew J., Slim Z., Fox S.A. (2023). Dynamic real-time optical microscopy of oral mucosal lesions using confocallaser endomicroscopy. J. Oral Pathol. Med..

[127] Horgan C.C., Jensen M., Chiappini C., Vercauteren T., Cook R., Bergholt M.S. (2022). Hybrid confocal Raman endomicroscopy for morpho-chemical tissue characterization. Biomed. Opt. Express.

[128] Winnand P., Ooms M., Heitzer M., Lammert M., Hölzle F., Modabber A. (2023). Real-time detection of bone-invasive oral cancer with laser-induced breakdown spectroscopy: A proof-of-principle study. Oral Oncol..

[129] Abdel Razek A.A.K., Khaled R., Helmy E., Naglah A., AbdelKhalek A., El-Baz A. (2022). Artificial Intelligence and Deep Learning of Head and Neck Cancer. Magn. Reson. Imaging Clin. N. Am..

[130] Rodriguez-Diaz E., Jepeal L.I., Baffy G., Lo W.K., MashimoMD H., A’amar O., Bigio I.J., Singh S.K. (2022). Artificial Intelligence-Based Assessment of Colorectal Polyp Histology by Elastic-Scattering Spectroscopy. Dig. Dis. Sci..

[131] Daoust F., Nguyen T., Orsini P., Bismuth J., de Denus-Baillargeon M.M., Veilleux I., Wetter A., Mckoy P., Dicaire I., Massabki M. (2021). Handheld macroscopic Raman spectroscopy imaging instrument for machine-learning-based molecular tissue margins characterization. J. Biomed. Opt..

[132] Giordano S., Takeda S., Donadon M., Saiki H., Brunelli L., Pastorelli R., Cimino M., Soldani C., Franceschini B., Di Tommaso L. (2020). Rapid automated diagnosis of primary hepatic tumour by mass spectrometry and artificial intelligence. Liver Int..

[133] Raghushaker C.R., Rodrigues J., Nayak S.G., Ray S., Urala A.S., Satyamoorthy K., Mahato K.K. (2021). Fluorescence and Photoacoustic Spectroscopy-Based Assessment of Mitochondrial Dysfunction in Oral Cancer Together with Machine Learning: A Pilot Study. Anal. Chem..

[134] Xie X., Yu W., Chen Z., Wang L., Yang J., Liu S., Li L., Li Y., Huang Y. (2023). Early-stage oral cancer diagnosis by artificial intelligence-based SERS using Ag NWs@ZIF core-shell nanochains. Nanoscale.

